# 

**Published:** 1874-11

**Authors:** 


ESTABLISHED IN 1855.

Volume XII. | NOVEMBER—1874. | Number 8.

### ATLANTA
### Medical and Surgical Journal.

MONTHLY: THREE DOLLARS PER ANNUM, IN ADVANCE.

EDITORS:

ROBERT BATTEY, M.D.,

AND

W. F. WESTMORELAND, M.D.

*PAX ET SCIENTIA, SED VERITAS SINE TIMORE.*

ATLANTA, GEORGIA:

*DUNLOP, WYNNE & CO., BOOK AND JOB PRINTERS.*

1874.

